# Multi-objective optimization of multiple droplet impacts on a molten PCM using NSGA-II optimizer and artificial neural network

**DOI:** 10.1038/s41598-023-37712-x

**Published:** 2023-06-29

**Authors:** Shahin Faghiri, Parham Poureslami, Hadi Partovi Aria, Mohammad Behshad Shafii

**Affiliations:** 1 Department of Mechanical Engineering, Sharif University of Technology, Tehran, Iran; 2 School of Mechanical Engineering, College of Engineering, University of Tehran, Tehran, Iran; 3 Sharif Energy, Water and Environment Institute (SEWEI), Tehran, Iran

**Keywords:** Energy storage, Mechanical engineering, Engineering, Fluid dynamics

## Abstract

Embracing an interaction between the phase change material (PCM) and the droplets of a heat transfer fluid, the direct contact (DC) method suggests a cutting-edge solution for expediting the phase change rates of PCMs in thermal energy storage (TES) units. In the direct contact TES configuration, when impacting the molten PCM pool, droplets evaporate, provoking the formation of a solidified PCM area (*A*). Then, they reduce the created solid temperature, leading to a minimum temperature value (*T*_min_). As a novelty, this research intends to maximize *A* and minimize *T*_min_ since augmenting *A* expedites the discharge rate, and by lowering *T*_min_, the generated solid is preserved longer, resulting in a higher storage efficacy. To take the influences of interaction between droplets into account, the simultaneous impingement of two ethanol droplets on a molten paraffin wax is surveyed. Impact parameters (Weber number, impact spacing, and the pool temperature) govern the objective functions (*A* and *T*_min_). Initially, through high-speed and IR thermal imaging, the experimental values of objective functions are achieved for a wide range of impact parameters. Afterward, exploiting an artificial neural network (ANN), two models are fitted to *A* and *T*_min_, respectively. Subsequently, the models are provided for the NSGA-II algorithm to implement multi-objective optimization (MOO). Eventually, utilizing two different final decision-making (FDM) approaches (LINMAP and TOPSIS), optimized impact parameters are attained from the Pareto front. Regarding the results, the optimum amount of Weber number, impact spacing, and pool temperature accomplished by LINMAP and TOPSIS procedures are 309.44, 2.84 mm, 66.89 °C, and 294.98, 2.78 mm, 66.89 °C, respectively. This is the first investigation delving into the optimization of multiple droplet impacts for TES applications.

## Introduction

With an eye to diminishing carbon production, renewable energies have gained painstaking attention. However, variations in the supply of renewable energies have become a ubiquitous challenge, making thermal energy storage (TES) a consequential research topic^1^. In fact, TES plays a pivotal role in renewable energies as it bridges the gap between power supply and its requirement over the course of peak hours.

Among disparate approaches for storing thermal energy, latent heat thermal energy storage (LHTES) is of great significance and applicability by virtue of its noticeable storage capacity^2,3^. Phase change materials (PCMs) are extensively exploited in LHTES in view of not only high latent heat but trivial temperature changes during the phase change processes. Moreover, PCMs are widely employed in solar energy systems^4^, buildings^5,6^, desalination^7^, and thermal management of electronic devices^8^. However, the inherent defect of PCMs, low thermal conductivity, prolongs their melting (charge) and solidification (discharge) processes, restricting their applications. Metal foams^9^, expanded graphite^10,11^, nanoparticles^12,13^, or a combination of them^14,15^ have been widely utilized to enhance the thermal conductivity of PCMs. Applying the direct contact (DC) between the PCM and heat transfer fluid (HTF) is another innovative solution by which the heat transfer efficiency of PCMs may be critically enhanced, accelerating the melting and, notably, solidification procedures^16^.

Martin et al.^17^ employed the DC procedure to store cold. They asserted that flow rate, temperature difference, and droplet size are prevailing factors controlling the discharge process. Ameliorating heat transfer and sustainability performance, Hegner et al.^18^ investigated a direct contact LHTES system, employing ester as the PCM. They claimed that flowing HFT through the PCM results in tiny droplets, which improve the effective heat transfer area. Furthermore, they concluded that all melting and solidification processes lasted less than an hour despite their unoptimized system. Al Omari et al.^19^ scrutinized the influences of vibrations on the DC heat transfer between hot water and a heat sink comprised of a PCM. Comparing the results obtained under vibrations and static conditions, they observed a sharp enhancement in the cooling rate of hot water when the vibrational effects were brought to bear. Nonetheless, as the authors declared, further optimizations may be expected to improve the system's efficacy. Hosseininaveh et al.^20^ appreciably precipitated the charge and discharge rates of PCMs through the DC method, in which the HTF (acetone) boils within the paraffin and takes heat from it, bringing about the PCM solidification and HTF evaporation. Then, the acetone vapor condenses in the condenser, leading to numerous tiny HTF droplets. Following this, acetone droplets impinge on the surface of molten paraffin. The process continues so that the PCM is exhaustively solidified. They also indicated that, under the optimum situation, this approach strikingly lessens the discharge, charge, and total charging-discharging times. Utilizing the DC method between the HTF and PCM, Ramezani et al.^21^ decreased the overall discharge rate of PCMs from 2 h to 39 s. Similar to the previous scholarship, acetone droplets formed on the condenser impacted the molten PCM and caused it to solidify. Accordingly, the DC technique, through which the trouble of PCMs' slow discharge rate is ironed out, encompasses an interaction between the PCM and HTF droplets. This investigation strives to optimize the droplets' impact parameters and characterize the circumstances under which the PCMs’ discharge rate is further expedited in novel TES devices.

Disregarded in most surveys due to its sophistication, multiple droplet impacts (simultaneous, non-simultaneous, and successive) on liquid films, liquid pools, and solid surfaces have numerous applications, including spray cooling^22^, inkjet printing^23^, spray coating^24^, desalination^25^, and so forth. Liang et al.^26^ studied the simultaneous and non-simultaneous impact of a droplet pair at room temperature. According to their investigation, high Weber numbers (*We)* result in a central liquid sheet, which is a three-dimensional phenomenon. Moreover, increasing droplet vertical spacing (or time lag between droplets) in the non-simultaneous case brings about a decline not only in central liquid sheet height but in the spreading factor. They also found that the influence of vertical spacing declines as the *We* number increases. Employing the volume of fluid approach, Guggilla et al.^27^ studied the hydrodynamic and heat transfer characteristics of drop-on-drop impact on a hot surface. They maintained that the *We* number, Bond number, and Jakob number have an overriding influence on the spreading factor and evaporation dynamics of droplet impact. Guilizzoni et al.^28^ numerically surveyed multiple droplets simultaneously impacting the deep pool utilizing OpenFOAM® software. The results demonstrate that by increasing the impact spacing between droplets, the time evolution of craters would resemble that of a single droplet. Moreover, the shorter the impact spacing, the deeper the crater. Goswami and Hardalupas^29^ concentrated on the simultaneous impingement of two droplets on a dry surface. They claimed that the secondary droplets provoked by the uprising sheet splash are larger than those caused by a single droplet impingement. Kirar et al.^30^ experimentally examined the impact of two ethanol droplets on the ethanol pool at low impact velocity. They found that when the normalized distance between the droplet exceeds 3.2, the interaction between neighboring capillary waves is undermined, ending up with a partial coalescence. Generally speaking, there are overly few studies in the literature concentrating on the multiple droplet impact hydrodynamics and heat transfer.

Optimization is of great momentousness in the design of systems in light of its capability to address complex and practical problems^31^. As the term suggests, optimization refers to the process of finding the best values for an objective function based on a set of parameters. Single-objective optimization, in which only one objective function is considered, has a unique Pareto optimal solution. In contrast, multi-objective optimization (MOO)^32^ expands the idea of optimization by enabling the concurrent optimization of several individual objectives. MOO is a mathematical methodology that discovers diverse possibilities, all representing the optimal Pareto solution.^33^. Conducting MOO to minimize the PCM phase change time and maximize the exergy conversion, Zhang et al.^34^ suggested a combination of nanoparticles, metal foam, heat pipe, and fin to boost the efficacy of an LHTES unit. Taking heat transfer effectiveness and PCM melting time as the objective functions, Lin et al.^35^ performed MOO for a TES system. They raised heat transfer effectiveness by 34.0%. Huang et al.^36^ proposed a MOO method utilizing a genetic algorithm to reduce entropy generation and the upfront cost of geothermal ground heat exchangers. They lessened the operation cost of the system by 9.5%. Liang et al.^37^ explored the influences of PCM heat transfer improvement and geometric optimization on the overall storage performance. The results reveal that increasing the ratio of tube length to tube diameter instigates an increment in the effective energy storage ratio. Peng et al.^38^ conducted numerical optimization of patterned metal fins by adjusting their dimensions to optimize the convection-controlled and conduction-controlled regions during the charging process. The results demonstrated significant improvements in charging performance, which ranged from 42.7% to 63.7%, depending on the optimization arrangement and heating temperature. Given the literature, there are few surveys on the optimization of TES systems composed of PCM^39^. Also, few studies have been focused on optimizing the droplet impact process^40^, entailing researchers to accomplish thorough investigations in this field. Moreover, no study has been conducted to optimize the impact parameters of liquid droplets so that the phase change time of the PCM is reduced.

In conclusion, the continuous impact of multiple HTF droplets on the PCM surface in the DC technique, solidifying the entire PCM expeditiously, provides an ingenious solution for the PCMs' meager thermal conductivity. As a representative of genuine DC energy storage units, the simultaneous impingement of a droplet pair on the PCM surface is considered here so that the interaction between neighboring droplets can also be taken into account. Once impacting the molten PCM pool, droplets absorb heat from the pool and evaporate, which solidifies a portion of the paraffin and lessens its temperature. Thus, in this research, solidified PCM area (*A*) and the minimum pool temperature value after the impact (*T*_min_) are the objective functions characterized by high-speed and IR thermal imaging, respectively. Three design parameters affecting the objective functions are the *We* number, horizontal impact spacing (*d*_h_), and the PCM temperature before the impact (*T*). As a novelty, a MOO is implemented in this scholarship to characterize the impact conditions by which *A* is maximized, while *T*_min_ is minimized, which engenders a more sizeable and more stable solid, leading to a more expeditious charge and discharge processes (see Section "Experimental results"), which has been the main stimulant for conducting the research. To put it another way, the study strives to enhance the effectiveness of a TES unit working based on the direct contact between HTF and PCM, which is its primary significance. In this survey, the following stages are performed, respectively:

1. Immense experiments are conducted systematically for various design parameters so that the experimental values of the objective functions (*A* and *T*_min_) are procured.
2. Experimental data are employed to train the artificial neural network (ANN) to obtain two models for the objective functions.
3. The trained network is given to the multi-objective non-dominated sorting genetic algorithm II (NSGA-II) to accomplish MOO.
4. Eventually, the optimum impact conditions are attained from the Pareto front by final decision-making (FDM) approaches.

Multiple droplet impingement is an intricate phenomenon, which is largely due to the interaction between adjacent droplets; therefore, investigations allocated to this challenging topic are rare compared to studies surveying the single droplet impact. Hence, delving into the simultaneous impact of a droplet pair is one of the novelties of the research. Additionally, studies investigating multiple droplet impacts on a liquid pool are scarce. Also, intriguingly, this investigation embraces two simultaneous phase changes (i.e., paraffin solidification and ethanol droplet evaporation, which has not been explored previously. Moreover, to the best of the authors’ knowledge, there is no research in the literature concentrating on the optimization of droplet impact parameters for TES applications.

## Experimental method

### Selecting PCM and HTF

**Figure 1:**
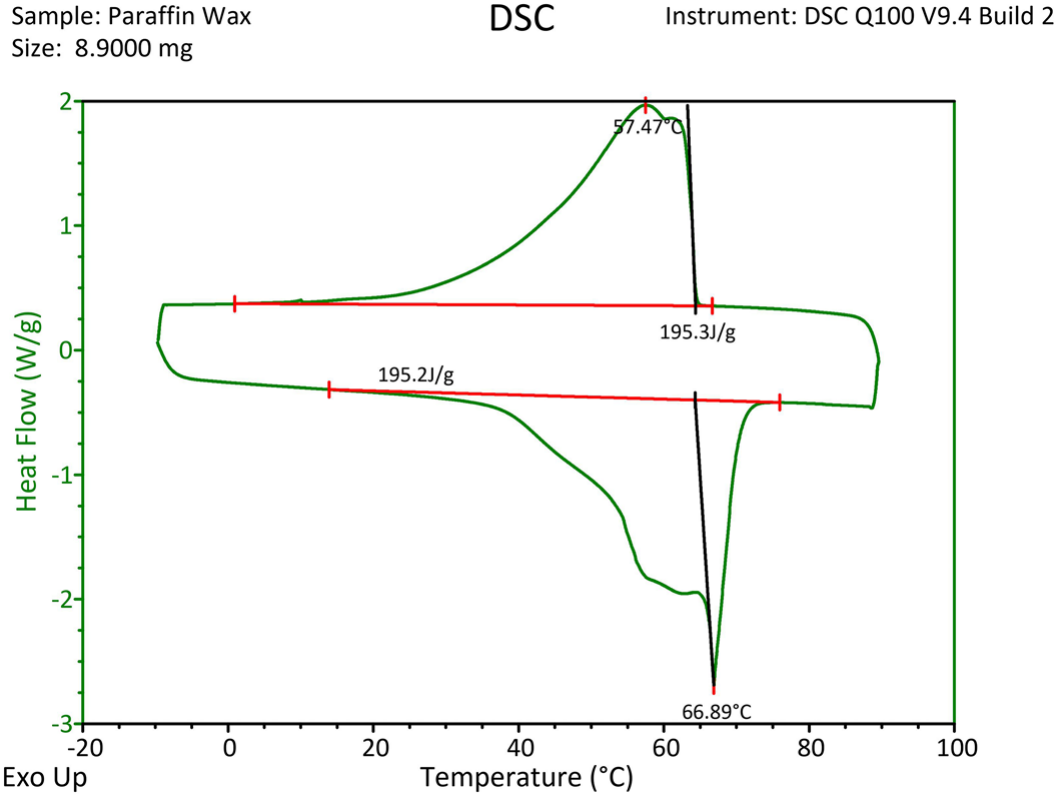
DSC analysis of granular paraffin wax, utilized as the PCM in the present study.

Paraffin wax was employed as the PCM due to its low cost, abundance, non-toxicity as well as high latent heat. The latent heat of paraffin and its phase change point were attained by the DSC experiment, whose results are shown in Fig. 1. Based on Fig. 1, the PCM solidification peak is 66.89 °C. Furthermore, the PCM’s thermal conductivity was specified by a KD2 pro instrument. Throughout the text, the terms PCM and paraffin are used interchangeably.

In the DC technique, the fluid saturation point should be lower than the PCM temperature; therefore, once contacting the PCM, the fluid evaporates and absorb heat from the paraffin so that the entire paraffin is solidified. Moreover, the HTF should be insoluble in the liquid PCM. Additionally, the density of PCM and HTF should be sufficiently different, making the separation between them uncomplicated^17^. Complying with the mentioned points, ethanol 96% was picked out as HTF for producing the droplets. Tables 1 and 2 summarize the properties of ethanol and PCM, respectively. It should be mentioned that the DSC analysis was also conducted for ethanol and paraffin mixture, whose results indicated that ethanol did affect paraffin properties negligibly as the ethanol is essentially insoluble in paraffin.

**Table 1:** Ethanol 96% properties.

Saturation point (°C)	Enthalpy of evaporation (kJ/kg)	*σ* (N/m)	*μ* (Pa.s)	*ρ* (kg/m^3^)	*c*_p_ (kg/kJK)
78.5	846.0	0.02255	0.0012	785	2.46

**Table 2:** Paraffin wax properties.

Solidification point (°C)	Enthalpy of fusion (kJ/kg)	Thermal conductivity at 80 °C (W/mK)	*c*_p_ at the liquid phase (kJ/kgK)
66.89	158.5	0.227	2.4

Since the paraffin temperature is higher than the ethanol saturation point, ethanol boils inside the PCM and subsequently evaporates; hence, the mode of heat transfer will be boiling whose heat transfer coefficient is sharply greater than convection; thus, applying a direct contact between the ethanol and paraffin may result in an exceeding increase in the melting and solidification rates of PCMs, which is desirable in TES, where paraffin is utilized as the storage material. Furthermore, DC heat transfer between ethanol and paraffin is useful for applications where the rates of charge and discharge should be monitored, which may be simply accomplished by varying the PCM to heat transfer HTF ratio. Also, this approach may be advantageous to cooling systems in which there is a limitation for the size of the system. Since the time of melting and solidification is overly short due to the high heat transfer rate caused by the boiling of HTF inside the PCM, numerous charge–discharge cycles may be fulfilled in a limited time, compensating for the low system size.

### Experimental setup

**Figure 2:**
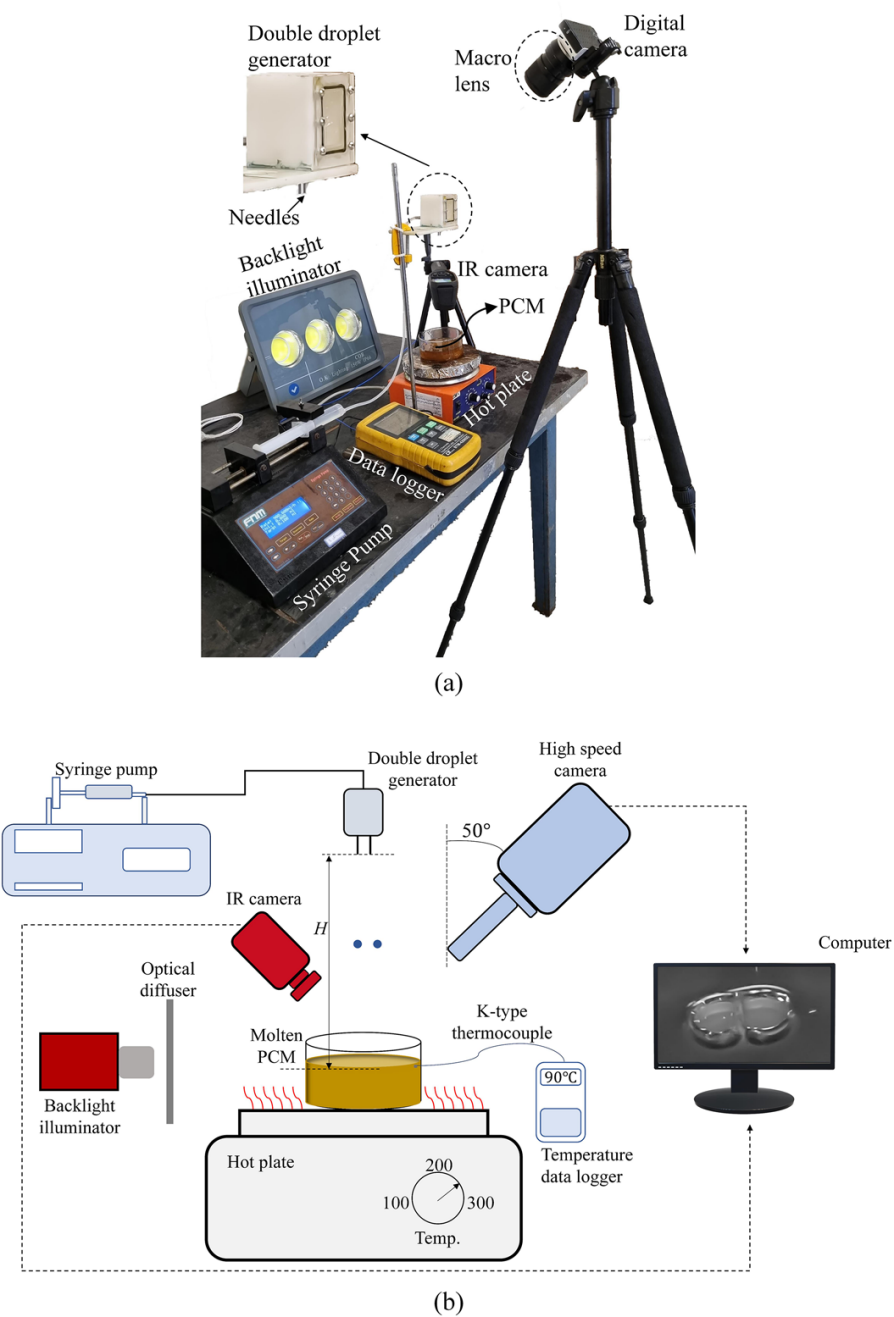
Experimental setup; (**a**) real image, and (**b**) schematic diagram.

Figure 2 demonstrates the experimental setup along with its schematic diagram. Initially, heated by a hot plate, paraffin reached a specific temperature (70, 75, 80, 85, 90, 95 °C), which was measured invariably using calibrated K-type thermocouples, connected to a data logger (BTM-4208SD). Then, the hot plate was deactivated. At this stage, by exerting a tiny force on a syringe utilizing a syringe pump, an ethanol droplet pair was separated from needles located at the height *H* (10, 15, 20, 25 cm). By varying the droplet release height (*H*), the impact velocity may be changed, which affects the Weber number (see Section "Image post processing"). Hence, the effects of the Weber number on the objective functions are examined by changing *H*. The values of *H* were chosen so that a wide variety of droplet impact dynamics is taken into account^41^; therefore, a comprehensive data set was exploited to train the artificial neural network. Furthermore, the *H* values were selected according to a TES unit performing based on the DC approach^20^. For droplet pair generation, two blunt-tip needles with an inner diameter of 1.6 mm were attached to the droplet generator. To survey the horizontal spacing effects on the objective functions, four droplet generator devices were constructed in which the distance between needles’ centers varies from 4.02 to 12.06 mm. Equipped with a Macro lens for magnification, a digital CMOS camera (Nikon 1 J4) was fixed 50° with respect to the vertical axis so that the PCM surface could be captured. To capture the details of the multiple droplet impact phenomenon, the digital camera shooting rate was set to 1200 Hz with 416 × 144 pixels in each image in which each pixel roughly corresponds to 0.11 mm (the calibration was conducted by an object with determinate dimensions). Moreover, having a resolution of 640 × 480 pixels, an IR camera (Seek Thermal) was employed to investigate the paraffin surface temperature distribution after the impingement. To carry out the shadowgraph method, an LED light source was used. In addition, an optical diffuser was used to monotonously diffuse the light. The paraffin container, whose outer diameter is 100 mm, has been constructed from Pyrex. Furthermore, the container’s dimensions were selected such that walls would not affect the impact dynamics^42^. The PCM height in the container was fixed at 30 mm in all experiments as well. Though ethanol is insoluble in paraffin and evaporates after the droplet impact, the paraffin was completely altered after each experiment to avoid any PCM thermal degradation and exclude the effects of ethanol on the paraffin properties. An exhaustive elucidation of the experimental setup has been provided in our previous research^41^.

### Image post processing

**Figure 3:**
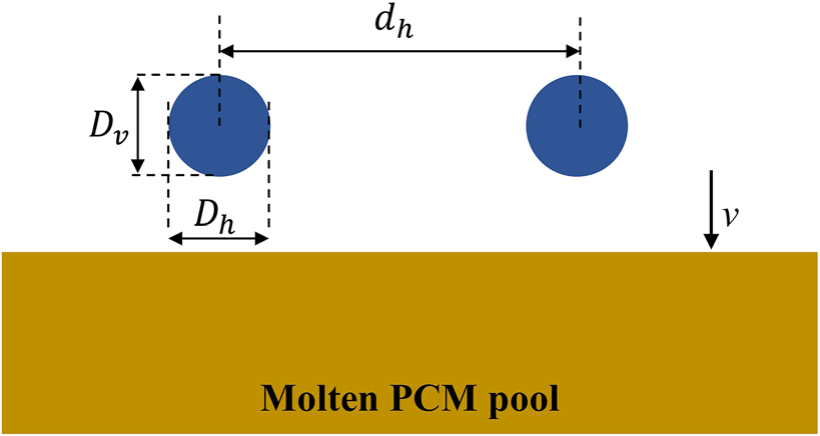
Defining impact parameters.

Impact velocity and initial droplet diameter were determined by pixel analyzing of the images. The initial droplet diameter was calculated as follows^43^:

1 $$D = \left( {D_{h}^{2} D_{v} } \right)^{\frac{1}{3}}$$

where ${D}_{v}$ and ${D}_{h}$ are the vertical and horizontal axes of the droplets as indicated in Fig. 3. Since droplets do not maintain a spherical shape during falling, ${D}_{v}$ and ${D}_{h}$ are not the same. By employing Eq. (1), the droplet diameter equals 2.68 ± 0.2 mm. Herein, the droplet diameter is supposed to be 2.68 mm for all experiments. The droplet impact velocity was obtained as follows:

2 $$v = \frac{{{\Delta }y}}{{{\Delta }t}}$$

where $\Delta y$ is the droplet displacement in the y-direction just before the impact, and $\Delta t$ is the time difference between the two successive images. It should be expressed that for computing the impact velocity and droplet diameter, the high-speed camera was fixed at 0°.

Additionally, the *We* number can be calculated as follows:

3 $$We = \frac{{\rho v^{2} D}}{\sigma }$$

where *D* is the initial droplet diameter, *ρ* and *σ* are the ethanol density and surface tension, respectively, as given in Table 1. The experimental and theoretical impact velocities based on four falling heights (*H*) used in the study are provided in Table 3. Regarding Table 3, it is axiomatic that theoretical and experimental results are in apt agreement with the average error below 1%. Moreover, the impact nomenclature is schematically illustrated in Fig. 3, in which ${d}_{h}$ represents horizontal impact spacing and varies between 4.02 and 12.06 mm, as mentioned above. This parameter is nondimensionalized by the droplet diameter as follows^44^:

4 $$\overline{{d_{h} }} = \frac{{d_{h} }}{D}$$

where $\overline{{d }_{h}}$ is defined as dimensionless horizontal spacing, whose values alter from 1.5 to 4.5.

**Table 3:** Experimental and theoretical impact velocity along with the *We* number based on four different falling heights used in this study.

*H* (cm)	${v}_{exp}$(m/s)	${v}_{theory}$ $(\sqrt{2gH}, m/s$)	*We*
10	1.38	1.40	179
15	1.71	1.72	275
20	1.99	1.98	373
25	2.21	2.21	464

**Figure 4:**
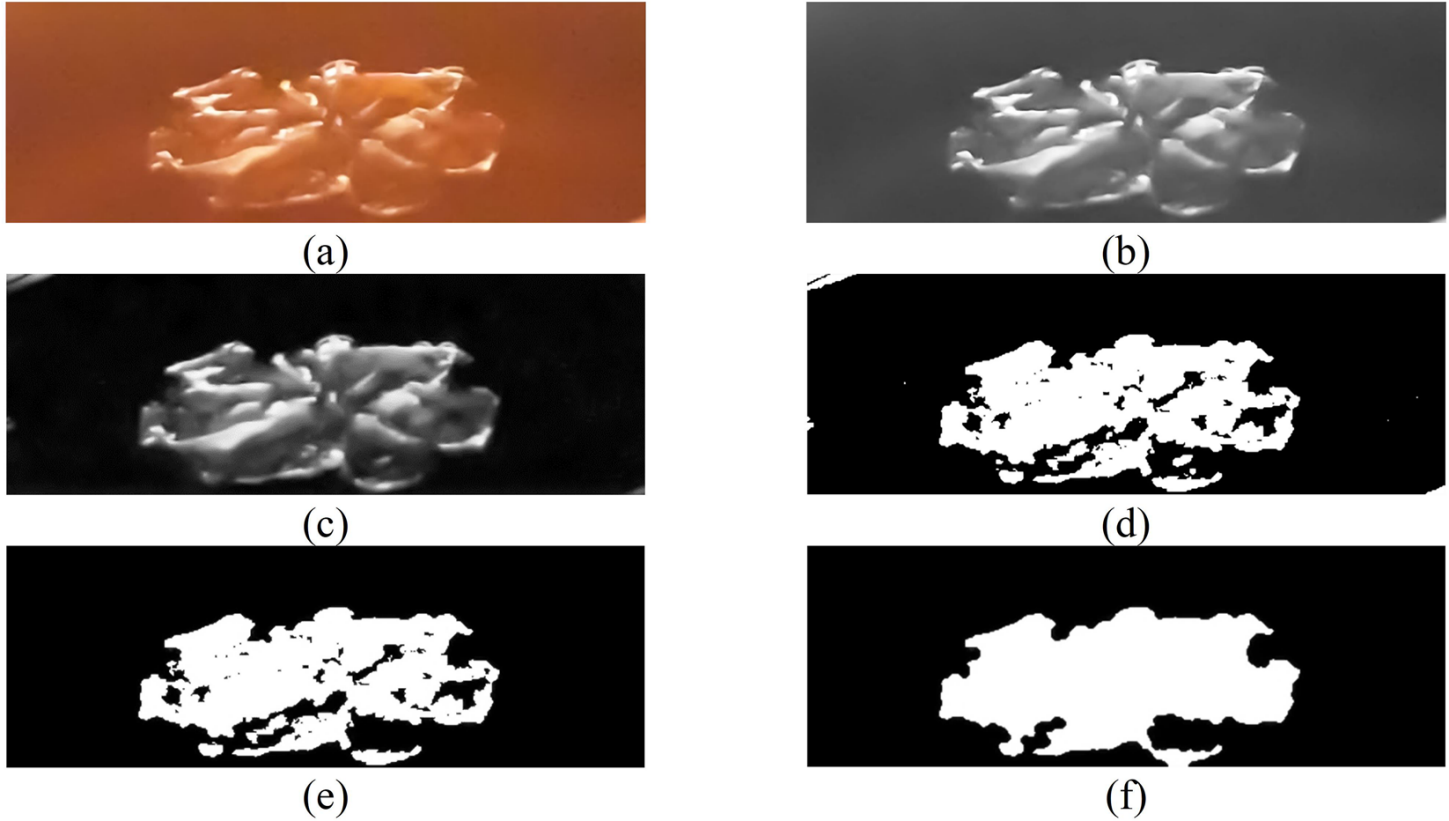
The post-processing stages implemented to determine the solidified PCM area; (**a**) original image, (**b**) grayscale image, (**c**) background subtraction, (**d**) binarized image and the edge diagnosis, the incorrect object that is occurred in the top left of the figure is unequivocal, (**e**) eliminating the tiny incorrect object happened in the previous step, and (**f**) the final image after filling the gaps; the area is calculated by this image. The impact conditions are *We* = 373, $\overline{{d }_{h}}$=2.5, and *T* = 70 °C*.*

Generated after two ethanol droplets impacting the paraffin surface, the solidified PCM area was determined by DMV software^45^, whose procedure is shown in Fig. 4. In the first stage, Fig. 4c, background subtraction was accomplished, accentuating the desired area. Subsequently, demonstrated in Fig. 4d, the grayscale image was converted to an 8-bit binary image via a user-defined threshold. At this step, the boundaries of the area were recognized. Then, minuscule erroneous objects, created in previous stages unintentionally (see Fig. 4d), were eliminated by a threshold, as shown in Fig. 4e. The border of the image can be omitted at this stage as well. Eventually, the fill holes option filled the gaps inside the region, ending up producing a well-defined zone whose area could be identified (Fig. 4f). The final values achieved from DMV were corrected by dividing $cos\left(\beta \right)$, in which $\beta$ is the tilted angle of the high-speed camera $\left(\beta =50^\circ \right)$.

### Uncertainty analysis

Inevitable errors in the current investigation chiefly originate from the measurement of the PCM temperature and droplet impact velocity. The paraffin temperature prior to the impact was attained by a k-type thermocouple, whose accuracy was 0.5 °C. Furthermore, the temperature distribution of the PCM surface was determined by an IR camera having an accuracy of 0.3 °C. In addition, data post-processing was implemented with an accuracy of 1 pixel, which corresponds to a 0.11 mm error. Thus, given the shooting rate of the camera (1200 fps), the impact velocity was calculated with an accuracy of 0.13 m/s. Based on Taylor’s theory, the uncertainty in the Weber number is as follows^46^:

5 $$\frac{\delta We}{{We}} = \sqrt {\left( {\frac{\delta D}{D}} \right)^{2} + \left( {\frac{2\delta v}{v}} \right)^{2} }$$

According to Eq. (5), the error in the Weber number calculation is, on average, 16.4% for different impact velocities. Table 4 recapitulates the accuracy of the experimental apparatus utilized in this survey.

**Table 4:** Uncertainty of experimental apparatus.

Apparatus	Error
Syringe pump	1 μl/h
High-speed camera	0.11 mm
k-type thermocouple	± 0.5 °C
IR camera	± 0.3 °C

## Experimental results

Two influential phenomena take place in the wake of ethanol droplet impact on the PCM pool: 1- absorbing heat from the PCM, ethanol droplets commence evaporating, and 2- the molten PCM is solidified. A couple of objective functions playing a momentous role in describing the mentioned incidences are the PCM solidified area (*A*) and the minimum PCM pool temperature value after the impact (*T*_min_), which are actually representative of the latent and sensible heat. Three design parameters, i.e., PCM pool temperature (*T*), impact spacing (*d*_h_), and *We* number influence the objective functions. The effect of these design parameters on objective functions will be scrutinized in this section. The experiments were carried out for four different *We* numbers (179, 275, 373, and 464), four different dimensionless impact spacing (1.5, 2.5, 3.5, and 4.5), and six various pool temperatures (70, 75, 80, 85, 90, and 95 °C); hence, a huge amount of experimental data (96 cases) was produced for the optimization purpose. To examine reproductivity and ensure accuracy, each case was repeated at least three times, and the presented data is the average of three cases.

**Figure 5:**
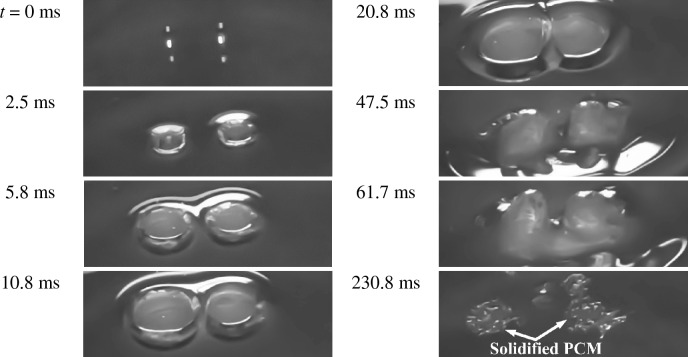
Time elapsed of solidified PCM area formation after simultaneous double droplet impact when *We* = 464, $\overline{{d }_{h}}$ = 3.5, *T* = 90 °C.

The process of solid formation on the PCM surface is revealed in Fig. 5 for the impact condition of *We* = 464, $\overline{{d }_{h}}$ = 3.5, and *T* = 90 °C, where *t* = 0 is the time at which the first droplet contact the PCM. Based on Fig. 5, craters are created shortly after the impingement (*t* ≈ 2.5 ms); then, they expand vertically downward and radially until reach the maximum depth and width (*t* ≈ 20.8 ms). When attaining maximum depth and width, craters rebound and two jets are ejected separately perpendicular to the paraffin surface (*t* = 47.5 ms), which evolute to achieve maximum height (*t* ≈ 61.7 ms). Next, jets descend, and their heights diminish, ending up forming solidified PCM areas (*t* = 230.8 ms) on the pool surface. The detailed examination of impact dynamics is far beyond the scope of the present study.

### PCM temperature effect

**Figure 6:**
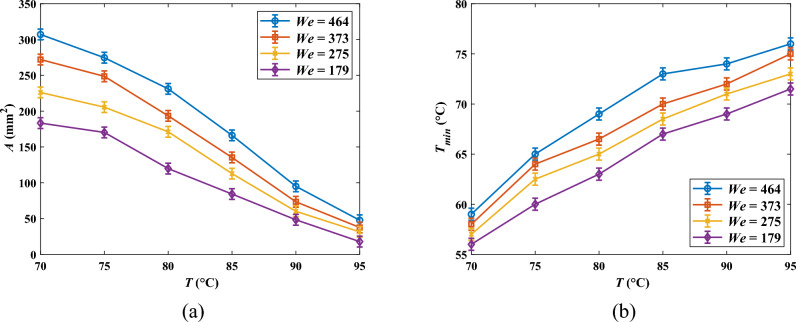
Solidified PCM area (*A*) and minimum pool temperature value after the impact (*T*_min_) as a function of PCM temperature (*T*) for different *We* numbers at $\overline{{d }_{h}}$ = 2.5; (**a**) *A*(*T*), and (**b**) *T*_min_ (*T*).

Figure 6 represents the influence of paraffin pool temperature (*T*) on the solidified PCM area (*A*) and the minimum pool temperature values after the impact (*T*_min_) for various *We* numbers at $\overline{{d }_{h}}$ = 2.5. Based on Fig. 6a, it is obvious that *A* increases exceedingly as temperature diminishes for all *We* numbers. Indeed, when *T* is high, the major portion of heat extracted by ethanol droplets is sensible heat, lessening the paraffin temperature; therefore, the insignificant part of the absorbed heat is allocated to the PCM solidification, leading to a small solidified area. That is why the area at *T* = 95 °C is quite small. Conversely, when the PCM temperature is sufficiently near to the freezing point, the remarkable portion of the heat absorbed by droplets is dedicated to the phase change and the negligible one is utilized to reduce the temperature up to the solidification point in the form of sensible heat, engendering a sizeable solidified area.

Moreover, regarding Fig. 6b, it is lucid that *T*_min_ decreases when the pool temperature dwindles. At low pool temperatures, the chief part of the absorbed heat causes the phase change and curtails the created solid temperature well below the freezing point. In contrast, at high pool temperatures, *T*_min_ is rather close to the phase change point, indicating that the solid zone can be wiped out promptly after the formation by absorbing heat from the pool, which is at a higher temperature. Consequently, not only does lessening the temperature provide a larger solidified area, but it reduces its temperature as well, which provokes a more “stable” solid region preserving for a longer period. In contrast, when *T*_min_ is high, the created solid swiftly melts. It should be stated that although at *T* = 95 °C minimum temperature value does not necessarily reach the solidification point of 66.89 °C, the solidified PCM area was observed for this temperature since based on the DSC analysis the solidification process is actually initiated at about *T* = 75 °C (see Fig. 1).

**Figure 7:**
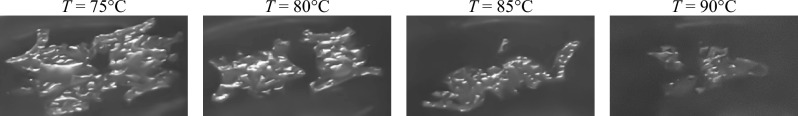
Solidified paraffin produced on the pool surface for various pool temperatures at *We* = 373 and $\overline{{d }_{h}}$=3.5.

**Figure 8:**
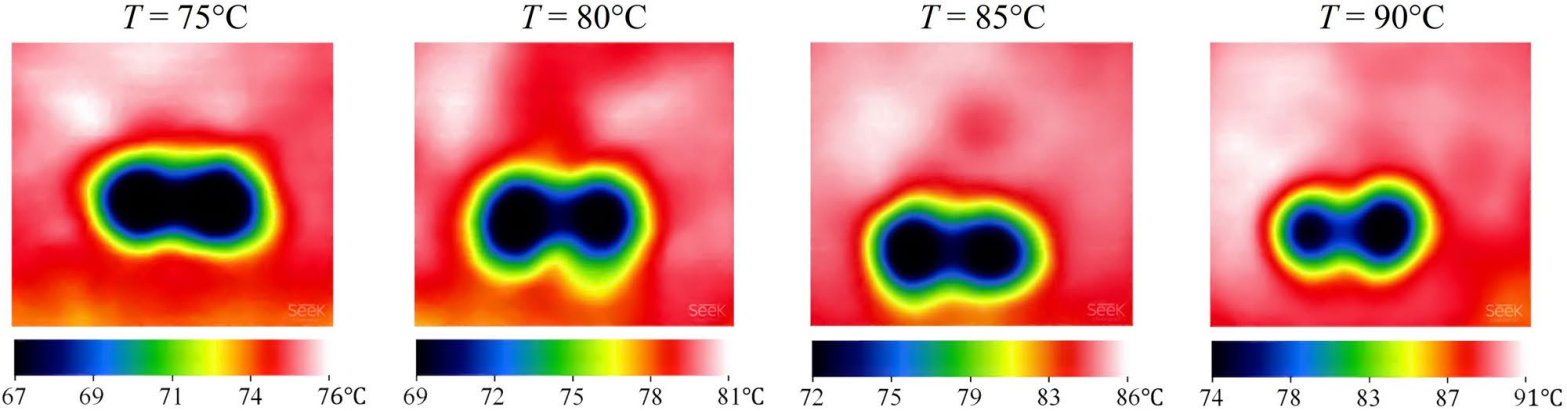
IR images of the PCM surface after the simultaneous impact of two droplets for various pool temperatures at *We* = 373 and $\overline{{d }_{h}}$ = 3.5.

Figure 7 indicates the influence of PCM temperature on *A* for several pool temperatures when *W*e = 464 and $\overline{{d }_{h}}$= 3.5. Unambiguously, the less the temperature, the more sizeable the solidified area. For instance, *A* at *T* = 90 °C is 83.5 mm^2^, while that at *T* = 80 °C is 218.7 mm^2^; thus, an 11.1% reduction in temperature brings about a dramatic increment of 161.9% in *A*. Additionally, achieved by IR thermal imaging, Fig. 8 demonstrates the influence of pool temperature on *T*_min_ in the mentioned impact conditions. As can be seen, the maximum temperature in each contour is slightly higher than the reported values of *T* since reported pool temperatures are indeed the average value measured by the thermocouple and slightly lower than the maximum temperature. Based on Fig. 8, by reducing the temperature from 75 °C to 90 °C, the minimum temperature is increased by roughly 11.0%.

### Weber number effect

As indicated in Fig. 6a, augmenting *We* raises *A* for all pool temperature values. Actually, increasing the *We* number enhances the droplets’ inertia, which facilitates the spreading of the droplets onto the pool surface after the impact. Hence, the effective heat transfer surface between the PCM and the fluid is ameliorated, which instigates the solidified PCM area growth. For instance, *A* at *We* = 275 is, on average, 25.7% higher than that at *We* = 179. Additionally, scrutinizing the graphs of Fig. 6, one can figure out that the influence of *We* on *A* becomes weaker when the paraffin temperature diminishes. Increasing the viscosity due to the temperature decrement accounts for the phenomenon since growth in the viscosity boosts viscous dissipation, impeding droplet spreading on the surface, engendering a lower heat transfer area.

By contrast, given Fig. 6b, increasing the *We* number gives rise to a gain in *T*_min_, which is unfavorable in energy storage units. In fact, when the *We* number is low, droplets do not spread aptly on the PCM surface, reducing the pool-droplet interaction. Hence, in low *We* numbers, droplets interact with an insignificant portion of the paraffin surface, and their enthalpy of vaporization is devoted to solidification and lowering the temperature of a small area. Consequently, at low *We* numbers, droplets solidify a small region and then lessen its temperature remarkably, provoking a low *T*_min_. In contrast, at high *We* numbers, droplets interact with a notable part of the PCM, meaning that the evaporation of droplets is allotted to heat exchange with a larger PCM surface. Accordingly, as causing a phase change of a substantial area, the heat absorbed by the droplets is not adequate to diminish the solidified area's temperature after the formation, engendering a higher *T*_min_. Accordingly, preserving for a longer time, the solid produced by low *We* numbers are more “stable” inasmuch as its temperature is far from the melting point. Conversely, being in higher temperatures, solidified PCM generated by high *We* number impacts have less stability and are annihilated shortly after the formation. This point is notably authentic when *T* > 85 °C.

**Figure 9:**
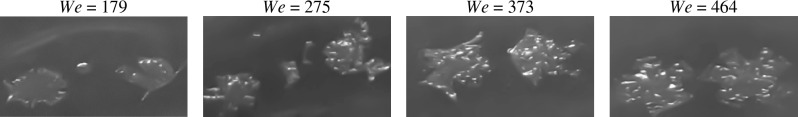
Solidified paraffin produced on the pool surface for various Weber numbers at *T* = 85 °C and $\overline{{d }_{h}}$=4.5.

**Figure 10:**
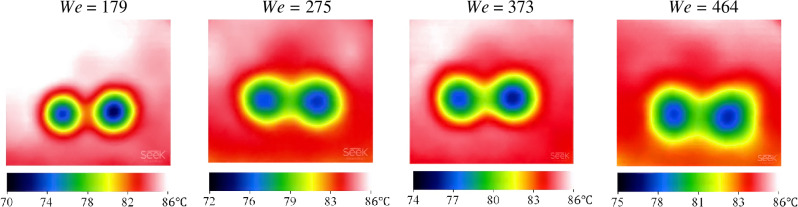
IR images of the PCM surface after the simultaneous impact of two droplets for various *We* numbers at *T* = 85 °C and $\overline{{d }_{h}}$ = 4.5.

Figure 9 reveals the effect of *We* on *A* when $\overline{{d }_{h}}$ = 4.5 and *T* = 85 °C. At *We* = 275 the solidified PCM area is 146.4 mm^2^, while this value reaches 206.6 mm^2^ for *We* = 464, implying that improving *We* by about 68.7% prompts a 41.1% enhancement in *A*. Figure 10 illustrates the temperature distribution of the paraffin surface after the impingement is in similar impact conditions. According to Fig. 10, reducing the *We* number from 464 to 179 has led to a 7% decrement in *T*_min_. Moreover, the impingement region can be differentiated conveniently in all cases due to the noticeable temperature reduction that occurred in the wake of the impact. Additionally, regardless of the *We* number, the most efficient heat transfer region has occurred in the impact crater zone since *T*_min_ has taken place in this region, which is consistent with Zhang et al.^47^.

### Impact spacing effect

**Figure 11:**
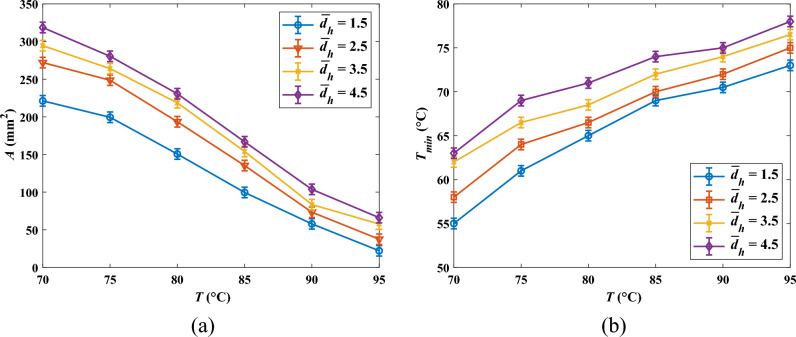
Variation of solidified PCM area (*A*) and minimum surface temperature (*T*_min_) against PCM temperature for four different horizontal impact spacing at *We* = 373; (**a**) *A* (*T*), and (**b**) *T*_min_(*T*).

Horizontal spacing has a consequential influence on the interaction between droplets, which in turn directly affects the objective functions of *A* and *T*_min_. Augmenting impact spacing substantially improves the solidified PCM area as demonstrated in Fig. 11a. Indeed, curtailing ${d}_{h}$ strengthens the droplet–droplet interaction and attenuates the droplet-PCM interaction, which diminishes the spreading area of the droplets. Therefore, less fluid contact with the paraffin, and the effective heat transfer area lessens, instigating a decline in *A*. For instance, according to Fig. 11a, A at $\overline{{d }_{h}}$ = 3.5 is, on average, 35% higher than that at $\overline{{d }_{h}}$ = 1.5. Additionally, the effect of horizontal spacing on *A* diminishes as the temperature decreases since lessening the temperature intensifies paraffin surface tension, which hampers droplets spreading on the surface and lessens the heat transfer area between the droplets and the PCM.

Conversely, concerning Fig. 11b, *T*_min_ is raised as the horizontal impact spacing increases, which is an undesirable factor in the TES system. At low impact spacing, since the spreading area is insignificant, droplets absorb heat from a small region of PCM, meaning that absorbed heat is dedicated to solidifying as well as lessening the temperature of a small zone. Consequently, in the case of shorter impact spacing, the created solid has a lower temperature. Hence, the shorter the impact spacing, the more stable the produced solid as it is maintained for a longer time.

**Figure 12:**
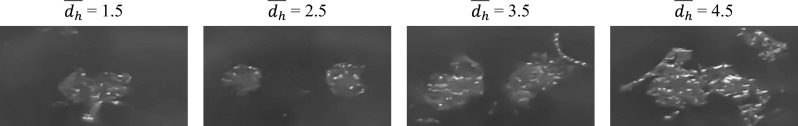
Solidified paraffin produced on the pool surface for various horizontal impact spacing at *We* = 464 and *T* = 85 °C.

**Figure 13:**
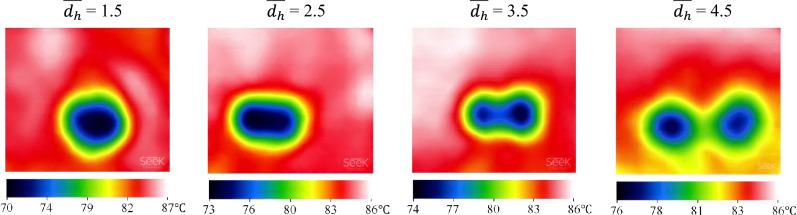
IR images of the PCM surface after the simultaneous impact of two droplets for various horizontal impact spacing at *We* = 464 and *T* = 85 °C.

The effect of horizontal spacing on the *A* is indicated in Fig. 12, in which *We* = 464 and *T* = 85 °C. Vividly, increasing impact spacing has engendered a dramatic improvement in *A*. IR images of the PCM surface are displayed in Fig. 13 in the mentioned impact parameters. Regarding Fig. 13, the impingement zone for $\overline{{d }_{h}}$ = 1.5 is smaller than that for $\overline{{d }_{h}}$ = 4.5. Therefore, the sensible and latent heat of the droplets absorbed from the PCM is assigned to a tiny region that not only solidifies it but also reduces its local temperature exceedingly after the phase change. As a result, an insignificant solidified PCM area with a lower temperature is accomplished for the lower values of impact spacing. In addition, the heat transfer in the impact crater has the foremost efficacy since *T*_min_ has occurred in this zone in all cases.

**Figure 14:**
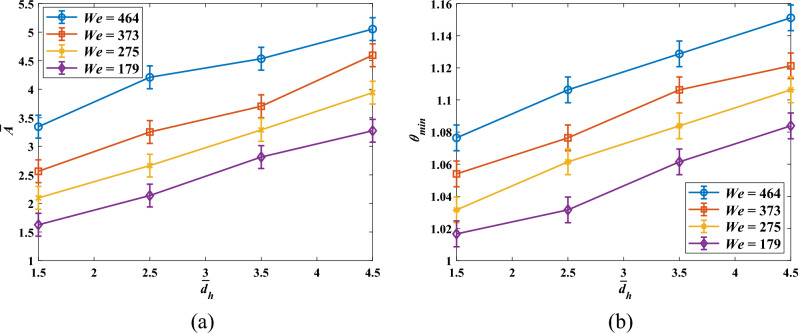
Variation of non-dimensional area ($\overline{A }$) and dimensionless minimum pool temperature value (*θ*_min_) against $\overline{{d }_{h}}$ for disparate *We* numbers at *T* = 90 °C; (**a**) $\overline{A } (\overline{{d }_{h}})$, and (**b**) ${\theta }_{min}(\overline{{d }_{h}})$.

Figure 14 represents non-dimensional solidified area ($\overline{A })$ and non-dimensional minimum pool temperature value after the impact (*θ*_min_) as a function of non-dimensional impact spacing for different Weber numbers are provided in, in which *T* = 90 °C. Evidently, $\overline{A }$ grows when horizontal spacing is boosted. Furthermore, $\overline{{d }_{h}}$ profiles shift upwardly as the *We* number is raised, indicating that the *We* number plays a critical role in the heat transfer characteristics of double droplet impingement. Therefore, as expected, the greatest $\overline{A }$ value corresponds to $\overline{{d }_{h}}$ = 4.5 and *We* = 464. On the other hand, *θ* increases as the *We* number and impact spacing grow, as described before.

Generally speaking, larger solids created on the pool after the impact are favorable in the DC approach as they hasten the discharge process; hence, more charge and discharge cycles can be achieved in a specific time. Moreover, the less *T*_min_, the more stable the solid. On the contrary, solids, whose temperatures are relatively high, are swiftly obliterated by absorbing heat from the molten PCM pool having a high temperature. Accordingly, the formation of a sizeable solid possessing a low temperature after the droplet impact is desirable in the DC procedure. However, as profoundly discussed in this section, though directly proportional to *A*, the Weber number and horizontal impact spacing are inversely proportional to *T*_min_. Therefore, by exploiting MOO, the current investigation strives to determine the optimum impact parameters through which *A* is maximized, and *T*_min_ is minimized, which enhances the efficacy of the TES unit. Designed under this optimum condition, a system may take advantage of a low volume since numerous charge and discharge cycles conducted in a particular time can compensate for a small volume.

## ANN and modeling

An artificial neural network (ANN) is a biologically-inspired computational model featuring numerous computational elements known as "neurons"^48^. In fact, ANN is a programming approach that acquires knowledge by observing data patterns. Initially, a collection of interconnected neurons is established to facilitate communication among them. Subsequently, a problem is formulated to be addressed by the network. The network undergoes iterative processes in an attempt to solve the problem and identify the correlation between input and output data. During the process, the network endeavors to minimize the error between the predicted and exact values. Eventually, by repeating this process, and with the help of thorough training examples and advanced computational power, ANN is capable of providing respond to various inquiries. The architecture of ANN comprises three layers, namely input, hidden, and output. It is worth mentioning that ANN has attracted the attention of researchers regarding modeling, predicting, or optimization problems such as optimizing energy systems, owing to its efficient capability in simulating and modeling nonlinear processes^49^.

**Figure 15:**
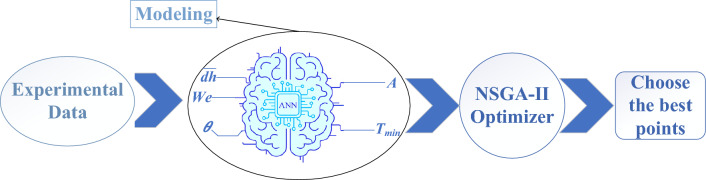
Flowchart of the modeling and optimization methodology.

Obtained in Section "Experimental results", the experimental values of *T*_min_ and *A* are considered as the inputs to the modeling problem serving as training data for the ANN . The trained network is then integrated into the multi-objective NSGA-II algorithm as a fitted function to perform MOO. In this regard, to evaluate the precision of the network in producing output results, a mean squared error (MSE) coefficient is utilized. Figure 15 represents the optimization methodology coupling the experimental and the neural network, as well as the neural network and NSGA-II. To train the network, the two variables of solidified PCM area and minimum pool temperature value after the impact ought to be dimensionless. By using the initial droplet diameter (*D*) and the paraffin phase change point, the aforementioned variables were nondimensionalized as follows^44^:

6 $$\begin{gathered} \overline{A} = \frac{A}{{\pi D^{2} }}, D = 2.68 {\text{mm}} \hfill \\ \theta_{\min } = \frac{{T_{\min } }}{{T_{s} }}, T_{s} = 66.89^\circ {\text{C}} \hfill \\ \end{gathered}$$

Also, the ranges at which design parameters vary are listed in Table 5.

**Table 5:** The range of the input variables changes.

Design parameters	bounds
$\overline{{d }_{h}}$	1–5
$\theta$	1–2
*We*	100–1000

Identical to ANN, the function fitting neural network (FFNN) bears three layers. The initial point of connection begins with the inputs, and each subsequent layer is linked to the preceding one, ultimately resulting in the production of the output layer^49^. The performance of the network is contingent upon the number of hidden neurons. The optimization of this number is achieved through a process of trial and error. Furthermore, two neural networks were trained to improve the accuracy of modeling for optimization purposes for $\overline{A }$ and *θ*_min_. Table 6 presents the neural network specifications applied to model the dimensionless solidified PCM area. For the purpose of training and testing the network, the data should be divided into three distinct datasets, including training, validation, and testing. During training, it is this set of data used to detect hidden features or patterns in the data. In each epoch, the model learns the features of the training data by feeding it the same training data repeatedly. At the same time, validation sets, separate from training sets, are used to validate the model during training. This validation process provides information that can be used to fine-tune the model's hyperparameters and configurations and determine whether the training is progressing in the right direction. The model is trained on the training set while being evaluated on the validation set at the end of each epoch, preventing the model from overfitting. A test set is then used to test the model's accuracy once it has been trained, providing an unbiased measure of the model's performance. In this concern, 15% of the data is used for validation, 15% for testing, and the rest for training. Furthermore, since the dataset is not noisy, Levenberg–Marquardt’s algorithm is employed in the current scholarship. According to this algorithm, training stops automatically when generalization does not improve, as shown by increasing the mean square error of validation samples.

**Table 6:** Variables used for dimensionless solidified PCM area's neural network.

Parameters	Value
Validation percentage	15%
Testing percentage	15%
Number of hidden layers	1
Number of hidden neurons	10
Training function	Levenberg–Marquardt
Validation checks	6

**Figure 16:**
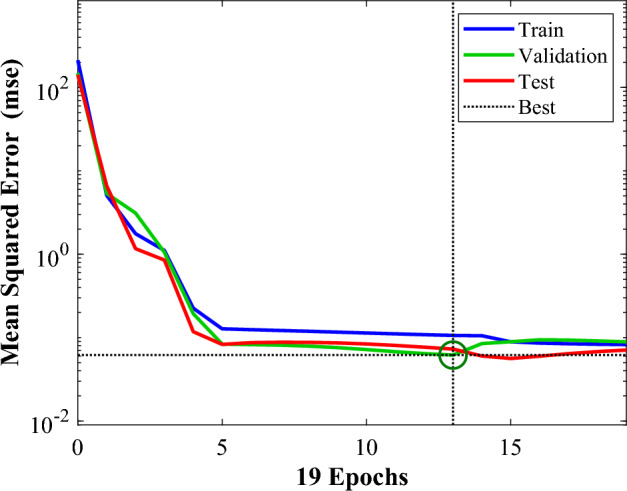
The ANN model's performance during training, testing, and validation for $\overline{A }$.

**Figure 17:**
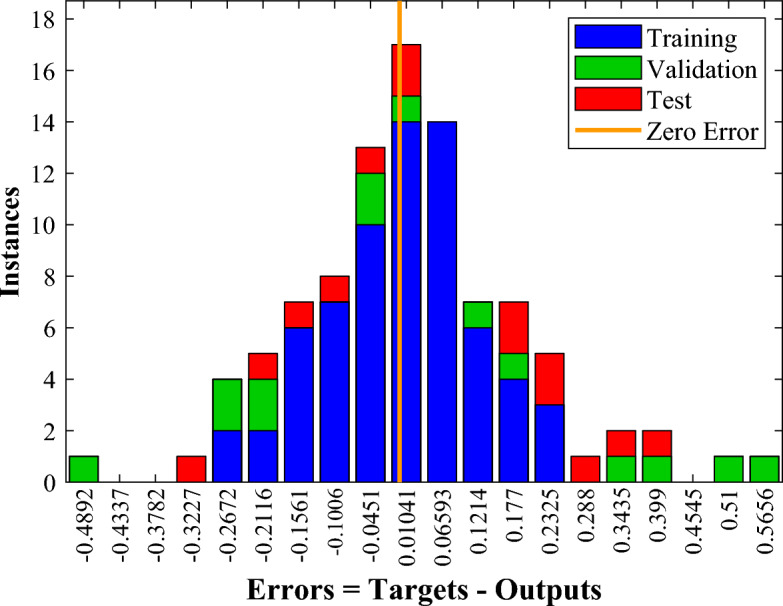
ANN model error histogram for $\overline{A }$.

**Figure 18:**
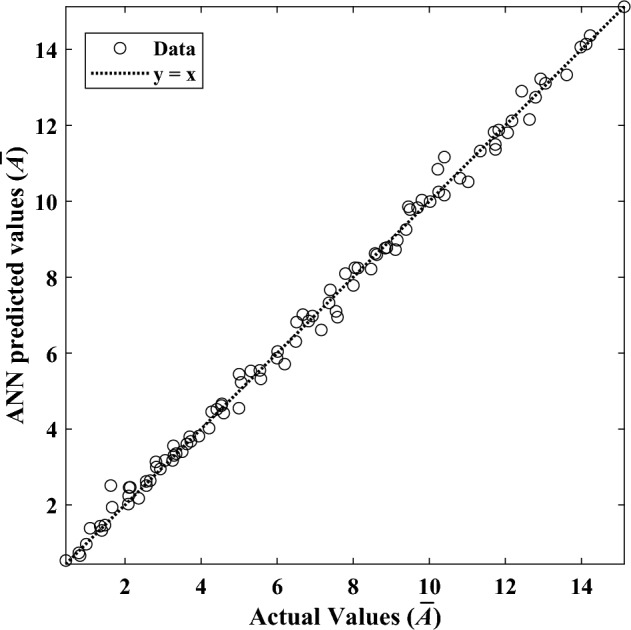
Results of the ANN prediction versus the actual data for $\overline{A }$.

By using the abovementioned parameter variables, the neural network was trained. Figures 16, 17 and 18 depict the neural network analysis of $\overline{A }$. According to Table 6 and Fig. 16, the validation process undergoes changes after 13 iterations, and the validation increment comes to a halt at iteration 19 to prevent overfitting during neural network iterations. That shows many iterations do not enhance the neural network's accuracy; in this scenario, iteration 19 is the best value.

Furthermore, Fig. 17 reveals the error histograms generated by the corresponding ANN model providing an exhaustive analysis of the model’s suitability and validity. The average relative error of this network to estimate the amount of $\overline{A }$ is equal to 2%, which indicates the acceptable accuracy of the trained network. In addition, to examine the efficiency of the neural network, the coefficient of determination (R^2^) is utilized. It is the value of R^2^ that determines how much the output results vary. Figure 18 provides a comparison between the ANN's output and the exact values. The closer the R^2^ value to one, the more accurate the estimate and, hence, the more reliable the trained network. This value for the dimensionless solidified PCM area's network is 0.98, which denotes high accuracy and reliability.

Another network is utilized to model the dimensionless minimum pool temperature value (*θ*_min_) as well. The specifications and parameters that are used in the neural network for *θ*_min_ are listed in Table 7.

**Table 7:** Variables used for dimensionless minimum pool temperature's neural network.

Parameters	Value
Validation percentage	15%
Testing percentage	15%
Number of hidden layers	1
Number of hidden neurons	5
Training function	Levenberg–Marquardt
Validation checks	6

**Figure 19:**
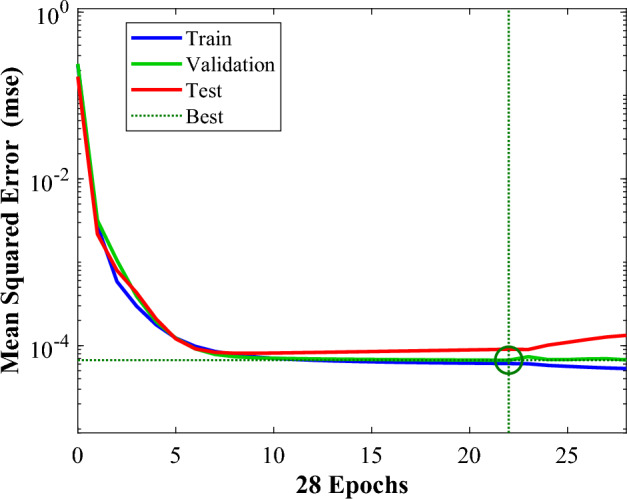
The ANN model's performance during training, testing, and validation for *θ*_min_.

**Figure 20:**
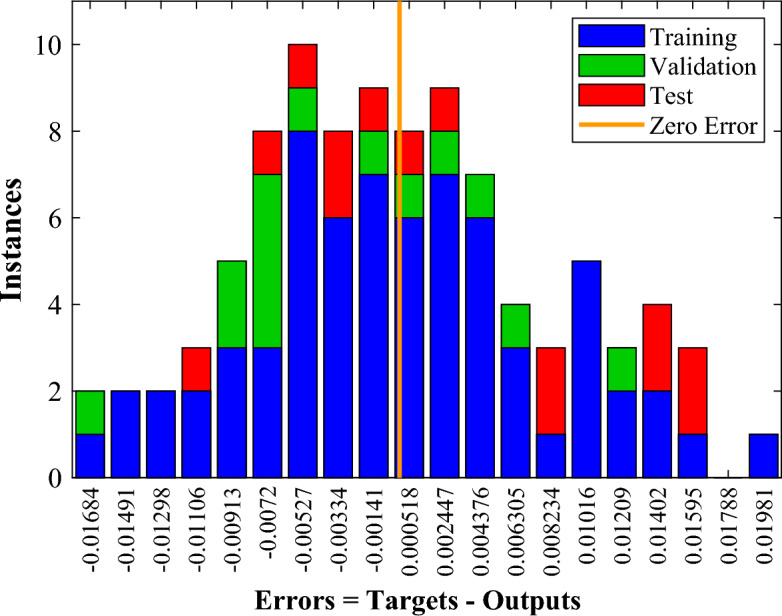
ANN model error histogram for *θ*_min_.

**Figure 21:**
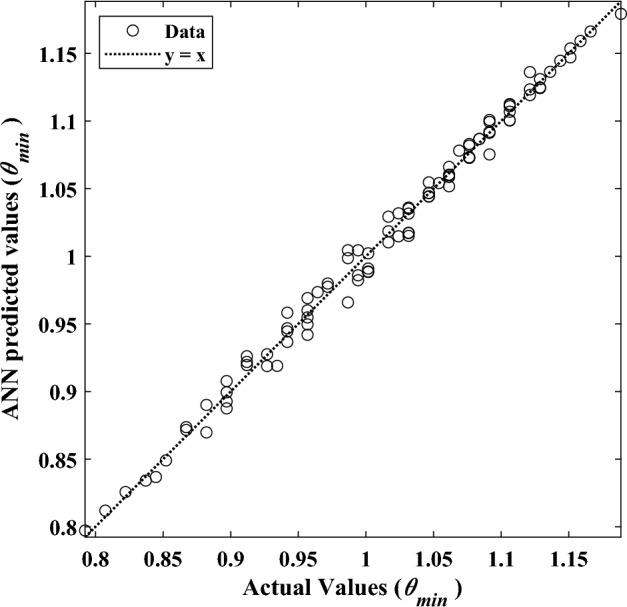
Results of the ANN prediction versus the actual data for *θ*_min_.

Figures 19, 20 and 21 represent the neural network analysis of *θ*_min_. According to Table 6 and Fig. 13, it can be concluded that, as in the previously trained network, the validation increment stops at iteration 28 to prevent overfitting after six iterations. Moreover, Fig. 20 shows the ANN model error histogram for *θ*_min_. According to Fig. 20, the average relative error for this network is 0.9%, demonstrating exceptional accuracy of ANN model. Moreover, based on Fig. 21, the network's coefficient of determination is equal to 0.99, testifying to the high accuracy of the trained network.

## Optimization

**Figure 22:**
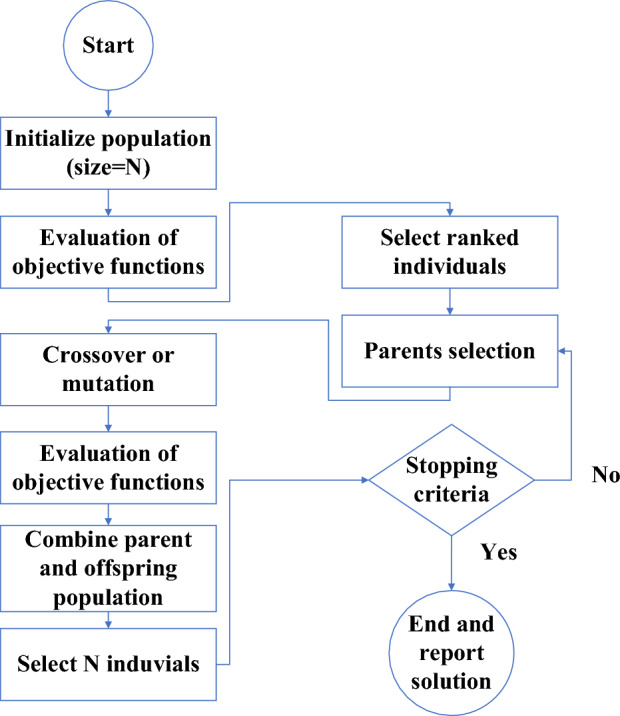
schematic of the NSGA-II flowchart^53^.

Optimization is a procedure leading to finding the optimal points of the system to minimize or maximize its performance; as it can be predicted, optimization is of great importance, having rendered many studies to focus on optimizing and designing energy systems to achieve higher performance^32^. In the meantime, the MOO approach is considered an efficient means of addressing real problems since systems in the real world usually contain several objective functions that are inconsistent with one another^50^. Real-world problems can have constraints and, in some cases, nonlinear objective functions, making them overly challenging to be solved. The mathematical representation of a MOO can be described as follows^51^:

7 $$F\left( x \right) = \left[ {F_{1} \left( x \right), F_{2} \left( x \right), \ldots , F_{k} \left( x \right)} \right]^{T}$$

which is subjected to:

8 $$\begin{gathered} G_{p} \left( x \right) = 0 \forall p = 1, 2, \ldots , P \hfill \\ H_{q} \left( x \right) \le 0 \forall q = 1, 2, \ldots , Q \hfill \\ \end{gathered}$$

in which the decision parameter vector is represented as x, and k indicates the count of objective functions. Equality and inequality constraints are represented by ${G}_{p} (x)$ and ${H}_{q} (x)$, respectively. Additionally, *P* and *Q* are the parity and constraint numbers, respectively^32^. Unlike single-objective optimization, in MOO problems, attaining a single optimal solution is impossible. Therefore, the Pareto optimal solution can be used. There is a concept called a non-dominant solution in solving MOO problems. In this case, a candidate solution for a MOO problem is referred to as a non-dominated solution when it improves the values generated by one or more objective functions of the problem while diminishing the quality of the values generated by other objective functions. These answers are known as "Pareto optimal solutions," which are equally suitable and considered as equal. For the purpose of optimizing several objective functions, in this research, the NSGA-II algorithm, an evolved genetic algorithm, is utilized due to its high efficiency and effectiveness in handling multi-objective problems. This is attributed to its fast crowded distance estimation strategy and non-dominated sorting procedure^52^. Figure 22 illustrates the schematic of the NSGA-II algorithms flowchart.

**Figure 23:**
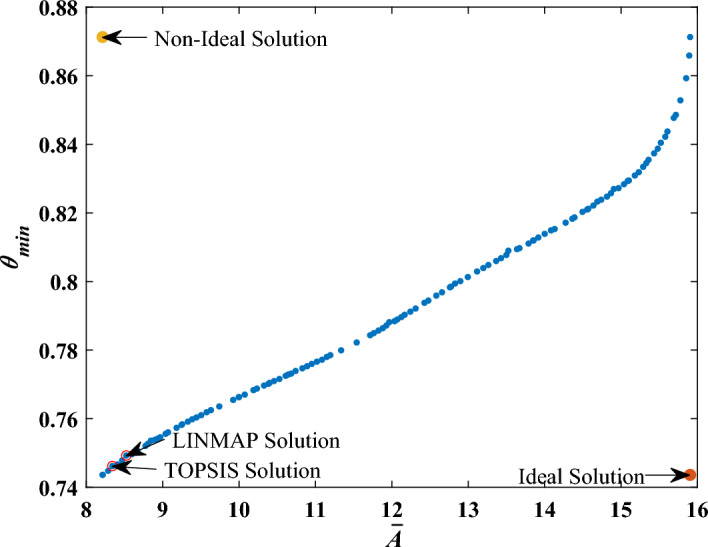
The Pareto front of the objective functions.

The ideal point (utopia) in optimization problems is where all the objective functions are in the most optimal possible state, and the non-ideal point is where all the objective functions are in the most non-optimal state. These points actually define the upper and lower limits. By considering these points, methods based on the final decision-makers (FDMs) can be used to overcome the problem of not providing a single optimal point in MOO problems. Herein, to attain a single optimal point on the Pareto frontier, two methods, the Linear programming technique for multi-dimensional analysis of performance (LINMAP) and the technique of order preference by similarity to an ideal solution (TOPSIS) are used. The governing equations of the LINMAP method are given as follows^53^:

9 $$\begin{gathered} ED_{i + } = \sqrt {\mathop \sum \limits_{s = 1}^{k} \left( {F_{is} - F_{S}^{ideal} } \right)^{2} } \forall i = 1,2, \ldots , R \hfill \\ i_{final} = i \in min \left( {ED_{i + } } \right) \hfill \\ \end{gathered}$$

here, R signifies the quantity of Pareto optimal solutions, while k represents the total number of objective functions. Equation (9) can be obtained by measuring the distance of each Pareto front point from the ideal point based on its Euclidean distance, while in the TOPSIS approach, in addition to considering the distance of the points from the ideal point, the distance from the non-ideal point is also taken into account. Thus, the TOPSIS method determines the closest point to the ideal point and the greatest distance from the non-ideal point with the following equations^53^:

10 $$\begin{gathered} ED_{i - } = \sqrt {\mathop \sum \limits_{s = 1}^{k} \left( {F_{is} - F_{S}^{non - ideal} } \right)^{2} } \forall i = 1,2, \ldots , R \hfill \\ ED = \frac{{ED_{i - } }}{{ED_{i - } + ED_{i + } }} \hfill \\ i_{final} = i \in max \left( {ED} \right) \hfill \\ \end{gathered}$$

where *k* and *R* are defined previously. Regarding Section "Experimental results", the parameters governing multiple droplet impact are the Weber number (*We*), paraffin temperature (*T*), and the impact spacing (${d}_{h}$). Therefore, these three variables are considered optimization parameters of the MOO problem. The objective functions of the problem are $\overline{A }$ and *θ*_min_ as well. As pointed out previously, this research aims to maximize $\overline{A }$ while minimizing *θ*_min_ so that each droplet impact leads to a large stable solid, which lessens the charge and discharge process. Figure 23 shows the Pareto front diagram of two-objective optimization by considering the mentioned objective functions.

Table 8 provides the results of MOO at the LINMAP and TOPSIS points.

**Table 8:** Ultimate optimum solutions procured by MOO.

FDM	$\overline{A }$	${\theta }_{min}$	*We*	*θ*	$\overline{{d }_{h}}$
LINMAP	8.524	0.749	309.444	1.000	1.061
TOPSIS	8.341	0.746	294.978	1.000	1.039

**Figure 24:**
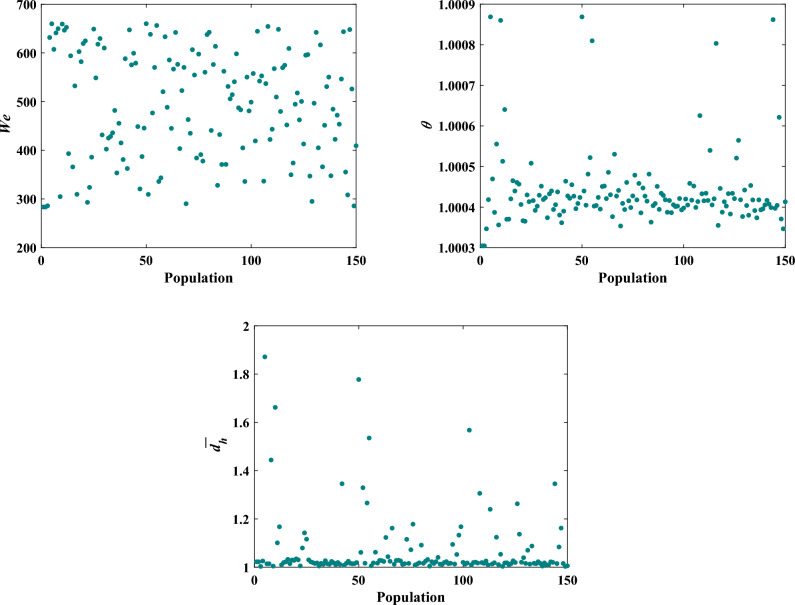
Scatter distribution of design parameters.

In Fig. 24, scatter distributions are displayed for various design parameters, showcasing the ideal range for the primary decision variables. As can be seen from Fig. 24, the large proportion of optimum points of nondimensionalized temperatures (*θ*) is in the range of 1.003 < *θ* < 1.005, and the horizontal distance is in the range of 1 < $\overline{{d }_{h}}$  < 1.2 as well. Nonetheless, the optimal points of *We* numbers are distributed across the entire range of 250–700.

## Conclusion

The multi-objective optimization (MOO) of a thermal energy storage (TES) unit working based on the direct contact of heat transfer fluid droplets (ethanol) and a phase change material (paraffin wax) was conducted for the first time. To carry out optimization, solidified PCM area (*A*) and minimum pool temperature value after the impact (*T*_min_) were considered as objective functions. Weber number (*We*), impact spacing (*d*_h_), and PCM pool temperature (*T*) have an outstanding effect on objective functions. Initially, experiments were implemented for $179\le We\le 464$, $4.02 \le {d}_{h}\le 12.06$ mm, and $70\le T\le 95^\circ C$. By altering impact parameters, a great amount of objective functions data was obtained employing high-speed and IR imaging as well as image post-processing. Subsequently, exploiting the artificial neural network (ANN), two models were fitted to each of the objective functions. In addition, it was shown that the accuracy and reliability of suggested models are excellent. Afterward, the trained networks were given to the multi-objective non-dominated sorting genetic algorithm II (NSGA-II) to perform multi-objective (MOO) optimization. Ultimately, through two disparate decision-making methods (FDMs), the optimum impact conditions were accomplished. The pivotal outcomes of the study can may be recapitulated as follows:

- Diminishing the PCM pool temperature results in a greater solidified area and a lower *T*_min_; both of which are advantageous to TES.
- While enhancing *A*, the increment of the *We* number raises *T*_min_. Accordingly, one should fulfill a trade-off between *A* and *T*_min_ when determining the Weber number.
- A decrement in horizontal spacing instigates a lower *T*_min_, while providing a smaller solidified area, entailing a trade-off between *A* and *T*_min_ during characterizing horizontal impact spacing.
- Final ideal solutions were obtained by LINMAP (*We* = 309.44, $\overline{{d }_{h}}$ = 1.06, $\theta$ = 1.00) and TOPSIS (*We* = 294.98, $\overline{{d }_{h}}$ = 1.04, $\theta$ = 1.00) form the Pareto front diagram.
- The maximum solidified PCM area determined by LINMAP and TOPSIS equals 192.34mm^2^ and 188.21mm^2^, respectively.
- The minimum pool temperature values attained by LINMAP and TOPSIS are 50.10 °C and 49.90 °C, respectively.

## Data Availability

The datasets generated during and/or analyzed during the current study are available from the corresponding author on reasonable request.

## List of symbols

$\overline{A}$: Dimensionless solidified PCM area $\left( {\overline{A} = \frac{A}{{\pi D^{2} }}} \right)$
*A*: Solidified PCM area (mm^2^)
*c*_p_: Specific heat capacity (kJ/kgK)
*D*: Initial droplet diameter (mm)
$\overline{{d_{h} }}$: The dimensionless horizontal impact spacing $\left( {\overline{{d_{h} }} = \frac{{d_{h} }}{D})} \right)$
$d_{h}$: Horizontal impact spacing (mm)
*H*: Falling height (cm)
*T*: PCM pool temperature (°C)
*T*_*min*_: Minimum pool temperature value after the impact (°C)
*T*_*s*_: Paraffin solidification point (°C)
t: Characteristic time (s)
*v*: Impact velocity (m/s)
*We*: Weber number $\left( {We = \frac{{\rho v^{2} D}}{\sigma }} \right)$

## Greek letters

*β*: The tilted angle of the high-speed camera (°)
*θ*: Dimensionless temperature $$\left( {\theta = \frac{T}{{T_{s} }}} \right)$$
*θ*_min_: Dimensionless minimum pool temperature value after the impact $\left( { \theta_{min} = \frac{{T_{min} }}{{T_{s} }}} \right)$
*ρ*: Ethanol density (kg/m^3^)
*σ*: Ethanol surface tension (N/m)

## Abbreviations

ANN: Artificial neural network
DC: Direct contact
FFNN: Function fitting neural network
HTF: Heat transfer fluid
IBF: Intermediate boiling fluid
IR: Infrared radiation
LHTES: Latent heat thermal energy storage
LINMAP: Linear programing technique for multi-dimensional analysis of performance
MOO: Multi-objective optimization
MSE: Mean squared error
NSGA II: Non-determined sorting genetic algorithm II
TES: Thermal energy storage
TOPSIS: Technique of order performance by similarity to an ideal solution
